# Coats'-like Response Associated with Linear Scleroderma

**DOI:** 10.18502/jovr.v17i1.10179

**Published:** 2022-01-21

**Authors:** Hassan Behboudi, Habib Zayeni, Asghar Haji-Abbasi, Zahra Moravvej, Ebrahim Azaripour, Yousef Alizadeh, Reza Soltani-Moghadam

**Affiliations:** ^1^Eye Research Center, Department of Eye, Amiralmomenin Hospital, School of Medicine, Guilan University of Medical Sciences, Rasht, Iran; ^2^Rheumatology Research Center, Razi Hospital, School of Medicine, Guilan University of Medical Sciences, Rasht, Iran

**Keywords:** Bevcizumab, Coat's Disease, Craniofacial, En Coup de Sabre, Scleroderma

## Abstract

**Purpose:**

To present a case of linear scleroderma known as “en coup de sabre” associated with Coats'- like response.

**Case Report:**

A 12-year-old boy presented with subacute painless vision loss in the ipsilateral side of the patient's en coup de sabre lesion. Ocular examination revealed vitreous hemorrhage with severe exudation of the posterior pole and telangiectatic vessels. Fundus fluorescein angiography indicated multiple vascular beadings and fusiform aneurysms with leakage which was consistent with a Coats'-like response. The patient was subsequently treated with intravitreal bevacizumab and targeted retinal photocoagulation. Twelve months' follow-up showed marked resolution of macular exudation with significant visual improvement.

**Conclusion:**

Physicians should be aware of the possible ophthalmic disorders accompanying en coup de sabre and careful ophthalmologic examinations should be performed in these patients. As presented in the current case, treatment with intravitreal anti-VEGF agents and laser photocoagulation may be a beneficial option for patients with coats'-like response.

##  INTRODUCTION

Linear scleroderma known as “en coup de sabre” (ECDS) is a form of localized scleroderma.^[1]^ The disorder presents with localized facial atrophy of the skin and the underlying tissue particularly in the frontoparietal area.^[1]^ En coup de sabre has been associated with a number of periocular and ocular manifestations. Periocular manifestations include enophthalmos and extraocular muscles and eyelids involvement. Ocular findings such as corneal alterations, cataract, iritis, and iris atrophy have also been reported.^[2,3]^ We report a case of ECDS presenting with decreased vision in the ipsilateral eye diagnosed as a Coats'-like response.

##  CASE REPORT

**Figure 1:**
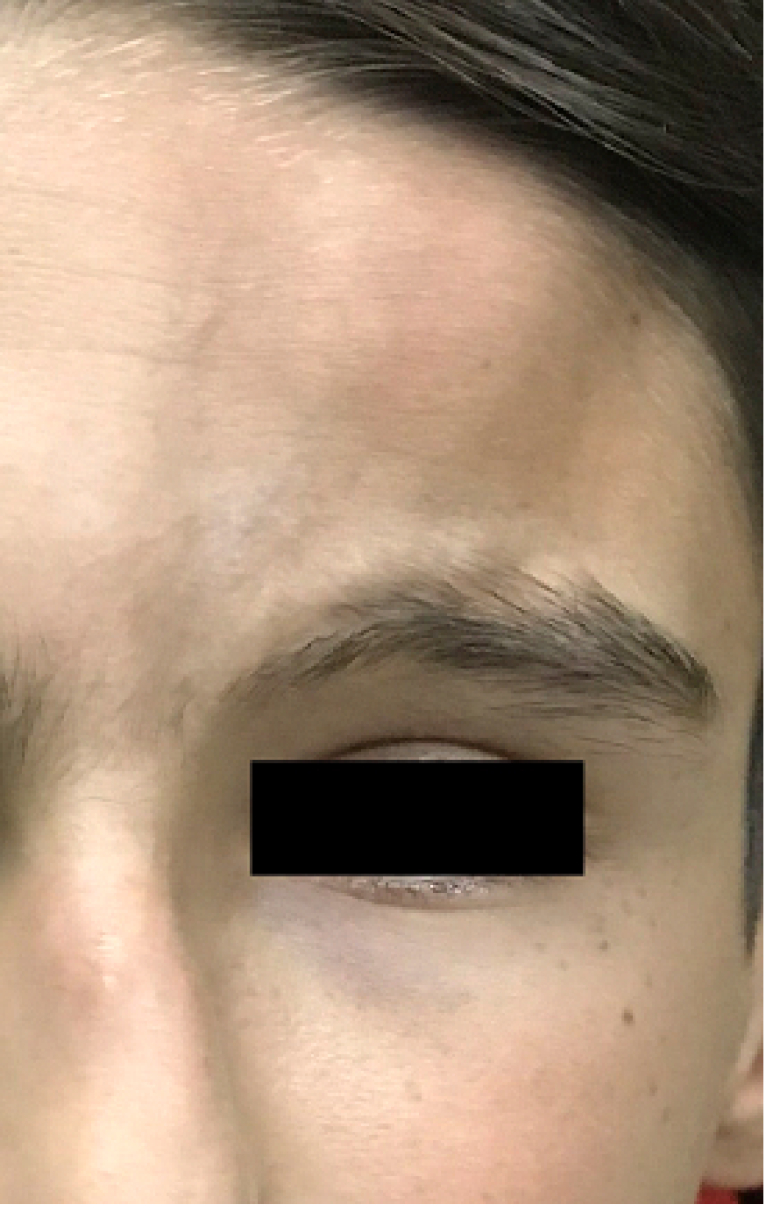
Photograph of the patient showing depressed left forehead skin lesion (en coup de sabre).

**Figure 2:**
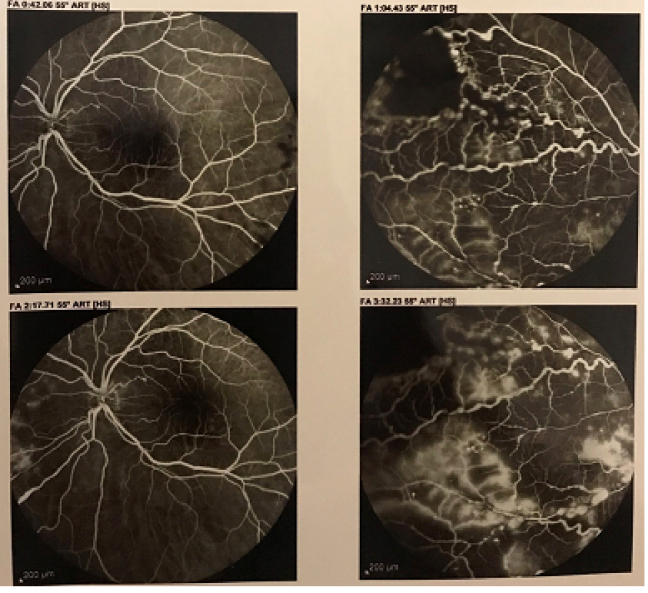
Fundus fluorescein angiography. Vascular tortuosity and fusiform aneurysms with leakage and non-perfusion areas in the mid-peripheral and peripheral regions.

A 12-year-old boy noticed painless vision loss in his left eye several days before. There was no history of visual disturbances and no family history of significant ocular disorders. He did not report any previous trauma and prior use of any medication. On physical examination, linear depressed scarring of the cutaneous and subcutaneous tissue of the left frontoparietal area was noted. This atrophic band of skin extended from the left eyebrow to the frontoparietal scalp [Figure 1]. His best-corrected visual acuity (BCVA) was 20/20 in the right eye and 20/200 in the left eye. The size of pupils was normal and there was no afferent pupillary defect. Ocular motility was within normal range. The intraocular pressure by applanation tonometry was 14 mmHg in both eyes. On slit lamp examination, the anterior segment was normal in both eyes. Funduscopy of the left eye showed vitreous hemorrhage with severe exudation in the posterior pole and telangiectatic vessels and saccular aneurysms in the mid-peripheral and peripheral retina. The optic disc was normal. The right fundus examination was unremarkable. Para-clinical evaluations with spectral-domain optical coherence tomography (SD-OCT) and fundus fluorescein angiography (FFA) were performed. SD-OCT of the left macula revealed intraretinal fluid and marked exudates [Figure 2]. FFA demonstrated vascular tortuosity and multiple beading and fusiform aneurysms with distinct leakage in the mid-peripheral and peripheral regions. Areas of capillary nonperfusion with no neovascularization were noted. The fundus findings were compatible with Coats'-like response. Corresponding rheumatologic consultation diagnosed his atrophic skin lesion as “en coup de sabre” (ECDS) a form of craniofacial linear scleroderma. Further examinations did not show any neurological signs or systemic involvement of scleroderma. Laboratory tests for antinuclear, anti-centromer, and Scl70 antibodies, erythrocyte sedimentation rate, and blood composition were normal.

**Figure 3:**
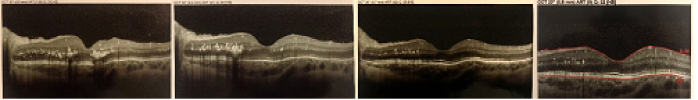
Macular optical coherence tomography at baseline, 3-month, 8-month, and 12-month visits (from left to right).

**Figure 4:**
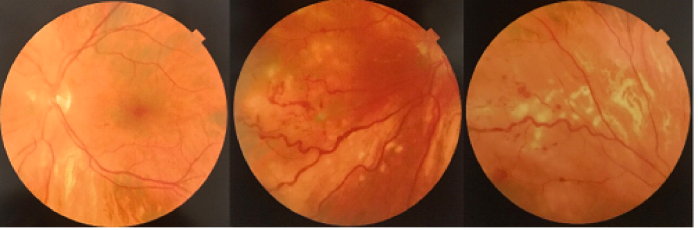
Fundus photograph at 12-month, showing reduced vascular tortuosity and beading.

The patient was scheduled for three monthly intravitreal injections of 1.25 mg/0.05 ml bevacizumab. On follow-up examinations, there was a significant decrease in macular exudation and vitreous hemorrhage. Targeted laser photocoagulation was performed over the abnormal retinal vasculature. Macular SD-OCT demonstrated significant reduction of intraretinal fluid and exudates [Figure 3]. At 12-month follow-up examination, BCVA improved to 20/25 in the left eye. Fundus examination and repeated FFA showed moderate resolution of vascular beading and tortuosity with no evidence of peripheral neovascularization [Figure 4]. The patient was scheduled for ophthalmic visits every three months. After one year of follow-up, we noted a loss of visual acuity (BCVA: 20/32) and moderate recurrence of macular edema and exudation. The patient was subsequently treated with one session of intravitreal injection of 1.25 mg/0.05 ml bevacizumab. He is currently under routine ophthalmic and rheumatologic observation.

##  DISCUSSION

Craniofacial linear scleroderma known as “en coup de sabre” (ECDS) presents with contraction and stiffness of the frontal or parieto-frontal area forming a depressed lesion in the skin and subcutaneous tissue.^[1]^ Various etiologies including trauma, radiotherapy, and autoimmunity have been proposed.^[4]^ En coup de sabre usually affects children in the first decade and is predominantly seen in females.^[5]^ Ocular manifestations is not common in localized scleroderma, however, it has been reported to occur in 14% of the patients with ECDS.^[3]^ Eyelid and adnexal involvement are the most common periocular abnormalities. Also, anterior segment inflammation is reported as the most frequent ocular manifestation.^[3]^

In the present case, a boy with ECDS presented with ipsilateral vision loss. The specific clinical picture and FFA were in favor of a Coats'-like response which refers to a fundus with the similar clinical appearance of Coats' disease in the setting of other ocular or systemic disorders. Coats disease is defined as idiopathic retinal light bulb telangiectasias with intraretinal and/or subretinal exudation without appreciable retinal or vitreal traction.^[6]^ The pathogenesis is believed to be related to the breakdown of blood–retinal barrier due to changes at the endothelial level and the presence of abnormal pericytes.^[7]^

To the best of our knowledge, there have been only five previous reports of this Coats'-like response in patients with ECDS.^[8,9,10,11,12]^ One of them resulted in exudative retinal detachment and severe vision loss in early childhood.^[8]^ Unlike previous reports, our patient regained nearly normal vision following appropriate treatment. We believe that treatment with intravitreal anti-VEGF agents and/or laser photocoagulation may be beneficial for patients with Coats' like response. This treatment may halt or at least delay progression of the retinal abnormalities.

It is of value to mention progressive hemifacial atrophy (Parry–Romberg syndrome) which is a hemifacial atrophy mainly below the forehead with an unknown etiology.^[13]^ Overlapping features of ECDS and ipsilateral hemifacial atrophy have been described in literature and it is thought that they may lie on the same spectrum.^[13]^ Coats'-like response has been reported in a number of cases with progressive hemifacial atrophy.^[14]^ The exact cause of this association remains undetermined; however, several theories have been suggested regarding the pathogenesis of scleroderma. The subclinical occlusive vasculitis can be caused by an inflammatory process with a probable autoimmune basis.^[15]^ Vascular abnormalities such as endothelial cells loss, increased vascular permeability, and defective angiogenesis have been recognized in linear scleroderma.^[16]^ It is hypothesized that systemic endothelial cell injury leads to the production of IFNα and subsequent tissue hypoxia and expression of VEGF.^[15]^ Intracranial vascular abnormalities have also been reported in patients with linear scleroderma. Gunness et al described an ipsilateral brain cavernoma in a patient with localized scleroderma on the frontal side of scalp.^[17]^

We presume that the vascular, inflammatory, and immunological processes involved may explain the vascular telangiectasia, dilatation, and leakage observed in Coats'-like response. Previous literature and the present case suggest that eyes as well as brain can be affected by linear scleroderma, which is commonly known as a limited skin disorder. Accordingly, we advise routine ophthalmologic examination including dilated funduscopy every three to four months in the first three years of presentation in patients with ECDS. Also, those presenting with visual complaints should be examined promptly. Pediatricians, dermatologists, and rheumatologists should be aware of the possible ophthalmic disorders associated with ECDS.

##  Financial Support and Sponsorship

Nil.

##  Conflicts of Interest

There are no conflicts of interest.
